# Utilizing the VeraFlo™ Instillation Negative Pressure Wound Therapy System with Advanced Care for a Case Study

**DOI:** 10.7759/cureus.903

**Published:** 2016-11-30

**Authors:** Rita K Driver

**Affiliations:** 1 Advanced Wound Healing Center, Jackson Purchase Medical Center

**Keywords:** therapy, veraflo, negative pressure wound therapy, chronic lymphedema, bioburden, instillation, dwell times, nutrition, necrotic, surgery

## Abstract

Background: Mankind has always suffered wounds throughout time due to trauma, disease, and lifestyles. Many wounds are non-healing and have continued to be challenging. However, utilizing advanced wound care treatments, such as negative pressure wound treatment with instillation and dwell time (NPWTi-d), has proven beneficial.

NPWTi-d is indicated in a variety of wounds, such as trauma, surgical, acute, pressure injuries, diabetic foot ulcers, and venous leg ulcers. Bacteria and bioburden interrupts wound healing by increasing the metabolic needs, ingesting, and robbing the necessary nutrients and oxygen.

Instillation therapy is the technique of intermittently washing out a wound with a liquid solution. The mechanism of action is instilling fluid into the wound bed, soaking for a determined time, loosening and cleaning of exudate, contaminants, and/or infection, removing fluid via negative pressure, thus promoting tissue growth.

Case study: The patient was diagnosed with a large lymphedema mass on the right upper thigh. Surgical removal of the lymphedema mass was indicated due to interference with quality of life. After a failed flap and surgical debridement, NPWTi-d with normal saline was implemented.

Results: The patient had excellent results, with obvious forming of red, beefy granulation, epithelization tissue development, and a cleaner, healthier wound bed. Settings for the NPWTi-d was 18 minutes dwell time, every 2.5 hours with a constant pressure of 125 mm/hg pressure.

Conclusion: The NPWTi-d demonstrated to be an instrumental treatment in supporting and stimulating healing. Early application of the treatment with normal saline as the instillation fluid prepared the previously failed wound for quicker healing.

## Introduction

Bacteria promotes an inflammatory environment, secreting harmful cytokines, which constricts blood flow, inhibits cell migration for tissue growth, and prevents wound healing/closure [1]. Bacteria and bioburden interrupts wound healing by increasing the metabolic needs, thus ingesting and robbing the necessary nutrients and oxygen needed for tissue repair and growth [1]. Increased amounts of bacteria and bioburden appeared to be a contributing factor in the failed surgical flap in the following case study. One reason in deciding to use the NPWTi-d was to remove the unwanted bacteria and for a cleaner wound bed and enhance granulation and wound healing.

The instillation of fluids is a technique of intermittently cleansing a wound. The instillation liquid the provider chose to use for this case study was normal saline. The mechanism of action is to intermittently and automatically instill the fluid into the wound beds, soak for a determined time to loosen and clean the tissue of exudates, contaminants, and/or infection, which is then removed via negative pressure into the collection canister. This treatment is called negative pressure wound therapy with instillation and dwell time (NPWTi-d). It is effective in treating complex wounds due to promoting tissue growth [2-3].

All wounds are prone to develop an infection. It is essential for good wound healing to prevent wound contamination and the invasion of foreign substances. Applying an airtight, sealed negative pressure dressing provides a protective covering for a period of time that deters contamination and invasion of such substances. The foam used as the dressing in the NPWTi-d is less hydrophobic, allowing more even distribution of the fluid to the wound bed for more effective soaking and dwell time, thus effective in wound cleansing.

Using negative therapy for the primary delivery of treatment has proven to have exceptional therapeutic results, along with automatic instillation to cleanse and wash the wounds from debris. The programmed unit delivers a preset amount of solution to be instilled, a set soak or dwell time, the cycle frequency, and provides continuous, prolonged, or intermittent settings. Studies have shown the use of NPWTi-d prepares the wound bed, removing infectious material, exudate, and debris, allowing healthy granulation tissue to form while preparing for wound closure. Wounds have a wide variety of factors during their healing process, which either continue to heal or the healing process stalls. Utilizing negative wound pressure therapy has a wide variety of healing mechanisms as shown in Table 1 [3-4].

**Table 1:** Mechanism of Action of Negative Wound Therapy

Decreases third space edema, allowing room for oxygen and nutrients to be delivered into the area which supplies to needed tissues.
Reduces wound exudate, thus decreasing the food source for bacteria.
Diminishes excessive inflammatory agents, which develop in wound stalls during the second state of healing, the inflammatory state. Inflammatory agents are known as matrix metalloproteases (MMPs), which decrease healing.
Promotes tissue stretching and contracting, known as macro and microstrain. This stimulates the release of essential growth factors to stimulate fibroblasts, increasing the construction of extracellular matrix (tissue structure/scaffolding), resulting in tissue granulation formation (pink or red, often beefy color, with small buds of tissue growth forming in the wound bed having an increased vascular supply and wound closure) [3].
Stimulates angiogenesis (blood vessel formation) [4].
Covers the wound, protecting the environment while maintaining a moist wound bed, essential for healing.

Wound care costs are a health care expenditure. These costs can increase significantly when there are difficult wounds that require more extensive treatments, such as surgery, wound care clinics, hospitalization, and advanced treatments. Utilizing nutritional support with negative wound therapy is effective to improve healing and recovery [5-6]. Even though negative pressure therapy may be considered a higher treatment cost, overall reports have shown cost savings, with fewer hospital stays/ readmissions, fewer surgeries, fewer dressing changes, shorter wound closure times, and increased limb salvage rates [6-7].

## Materials and methods

The product device used for this case study was the V.A.C. Ulta™ (NPWTi-d), (Kinetic Concepts Inc., an Acelity Co., Ireland), which provides conventional negative pressure, in addition to the instillation therapy. The wound bed is prepared for healing and closure by secondary or tertiary intensions. The programmed units provide continuous, prolonged, or intermittent negative pressures with a preset volume of fluid delivered into the wound bed for a set soak or dwell time and set negative pressure [3, 6].

Once these steps have occurred in promoting the environment for healing, along with increasing the blood flow and granulation (tissue development), then a process called resurfacing occurs when epithelization tissue (thin, delicate, pearly coloration that is present in the last stage of healing, the remodeling phase), marches from the margins, across the prepared wound bed, contracting and stretching until the wound is closed and healed [3].^ ^The treatment increases in secondary wound healing by using an open cell foam dressing placed into the wound bed. It is covered by a plastic drape, activated by subatmospheric negative pressure with an airtight seal, allowing for a moist wound bed [8]. This allows for decreasing the bacterial load, tissue edema, and wound tension, thus promoting angiogenesis/circulation [5]. Granulation tissue begins to form from the angiogenesis when the growth of new capillaries forms and reaches for the connective tissue. This allows healing by secondary intention when the wound is open and the gap is filled with granulation tissue before the epithelium is formed over the wound. This open area allows for the foam/dressing to be inserted into the wound bed. The initial NPWTi-d dressing was applied with sterile technique in surgery per the wound care APRN, Certified Wound Specialist (CWS), using a non-sting barrier skin prep to prepare the peri-wound area, circumferentially to the skin approximately 5 cm from the wound edge, then allowed to dry.^ ^It is essential for the adhesive dressing to maintain an airtight seal, preventing air leaks [9]. Air leaks are problematic and can disrupt and delay healing, making the wound prone to infection, due to secondary bacterial invasion. Sustaining an airtight system is critical to maintain optimum wound healing [7, 9]. Difficulties maintaining a wound vac seal are sometimes challenging, especially if the peri-wound skin area is not healthy. Table 2 provides recommendations, tips, and suggestions when applying the dressing and drape for a proper seal [3, 9-11].

**Table 2:** Tips for Negative Pressure Wound Vac Application and Proper Seal Management The following tips are based on the author's research and clinical experience [4, 10-12].

May use an adhesive removal pad to remove dressings, remove all adhesive residue. Be sure the adhesive pad is used onto the adhesive dressing, which is attached to the peri-wound skin. Pull or stretch the drape outward; this makes for easier removal.
Cleanse the peri-wound area; dry well. Avoid emollients, lotions, or products with fragrance, which can cause the drape not to seal.
May shave or clip hair, if problematic, for the drape sealing or staying sealed.
Premedicate for pain, if needed. The patient may assist with the dressing removal if potentially is easier on the patient.
Use Mepitel® One over bone, muscle, or tendon to protect.
Use the barrier film pad that is in each dressing kit for optimal drape adherence to the peri-wound skin.
For denuded peri-wounds from moisture problems, you may use the crusting technique to heal and protect the area, which is:
Start with clean dry skin.
Lightly apply ostomy powder onto the affected area, brush off excess (microscopically, the powder will sink down into the crevices). The area will look like all the powder is brushed off, but enough will still be present.
Light press non-sting barrier film (pad or spray) onto all the areas of skin breakdown with the ostomy powder to seal.
Repeat the process three times for moderate breakdown; may repeat five times for severe breakdown. It is important to brush all excess powder off and then seal with. LET DRY!
Perform the window pane technique to protect the peri-wound area. Apply a hydrocolloid or silicone-based barrier film around the wound perimeter, with good skin covered next to the wound margins.
May use stoma paste or moldable paste strips/putty in uneven areas to fill contours and help assist with moist areas.
For continued maceration or drainage from denuded skin, may consider painting the area with betadine. Check for allergies to betadine and shellfish.
Smooth folds and loose skin. For areas over joints, flex the joint area so when the joint area is moved, the dressing is placed in a stretched position to meet the moving and stretching needs to ensure adherence.
Use the barrier film pad over all of the margins/edges of the drape after applied to enough sealing the edges.
The VeraFlo™ therapy has an option for “fill-assist” or amount may be set by the programmer. Be sure the amount of fluid to be instilled saturates the black foam during each instillation cycle. The foam should rise somewhat during saturation and appear darker in color when saturated. If over-saturation occurs, the foam appears bulging and fluid is pooling around the edges of the wound or may leak out of the dressing. If this occurs, then lower the volume instilled and reseal the drape.
125 mm/hg is the suggested setting, except over bone, muscle, and tendon. May use lower setting until granulation tissue forms.
If the area of the wound vac application is on an upper or lower extremity, secure the wound vac dressing with Kerlix™ and Ace™ wrap [12].
Multiple dressing changes due to losing an airtight seal are time-consuming and costly. The adhesive drape for covering and sealing the foam dressing has moisture-resistant properties, which make the dressing adhere in moisture-ridden areas that are prone to increased perspiration and incontinence and where failure could be due to moisture [10-11].
A hydrocolloid was used to window frame the area for additional protection of the peri-wound area. VeraFlo™ dressing was inserted into the wound bed and into partial undermining areas. The VeraFlo™ dressing consists of a hydrophobic black foam designed for a more even distribution of instilled fluid [4, 12].
A moisture-resistant drape was applied to cover the wound for an airtight seal. The barrier film pad was applied along all the edges/margins of the applied drape and overlapping areas to seal micro leaks. The VeraFlo™ therapy settings were programmed to automatically deliver 66 cc’s of normal saline, with a dwell time of 18 minutes and the repeat cycle of 2.5 hours, with 125 mm/hg with continuous pressure. Adequate wound cleansing is crucial in the management of wound healing. Instituting the usage of fluid, such as saline, into the wound bed impedes cellular debris, dead tissue, elevated inflammatory markers, infection, and enzymatic constituents [12].

Negative pressure wound therapy is very established in the medical wound healing world. It can be used for a variety of wounds, including surgical wounds, traumatic wounds, pressure ulcers, diabetic foot ulcers, skin graft/flaps, chronic, acute, dehisced wounds, and partial-thickness burns [10, 12]. It utilizes subatmospheric pressure to enhance wound healing with an emphasis on increasing perfusion and the growth of granulation tissue.

Using highly computerized technology, the system can deliver intermittent or constant pressure into the wound bed with a durable open cell foam dressing with a SensaTRAC™ (therapeutic regulated accurate care) pad and sealed with an adhesive drape [7, 12]. Continued ongoing research and developments have led to advancements in wound treatments with the option of utilizing the instillation and dwell time feature [7].

"Without continual growth and progress, such words as improvement, achievement, and success have no meaning" - Benjamin Franklin [7].

The overall dressing is an airtight, water-impermeable, adhesive-sealed occlusive type drape. It is also oxygen and water vapor semi-permeable, which protects from water loss and contamination, allowing only gases to escape, while healing continues. The pumps have changed over time, with programmed profiles of prolonged, intermittent, or continuous suction and a range of pressure, indicating mm/hg. The Acelity V.A.C. Ulta™ has a dual program for either standard negative pressure treatment or NPWTi-d.

## Results

### Case presentation

The patient gave verbal and written permission for this publication. No identifiable information pertaining to the patient's identity was disclosed in the article.

The patient is a 52-year-old obese Caucasian male. The patient presented with a longstanding history of chronic bilateral lower extremity lymphedema, originally with the right upper extremity being the most affected area, with severe enlargement of the thigh region extending from the medial to the posterior aspect extending into a pannus type area. The lymphedema mass continued to worsen over the last 18 months with increased pain and tenderness. The diagnosis was chronic lymphedema with a large right upper thigh mass. Surgical excision of the right thigh lymphedema mass with anterior, medial, and lateral thigh flap closure covering a 30 cm x 30 cm area was performed. The wound was closed by primary intention, and the patient was discharged home. The patient is employed, has a sedentary lifestyle, and lives by himself.

**Figure 1:**
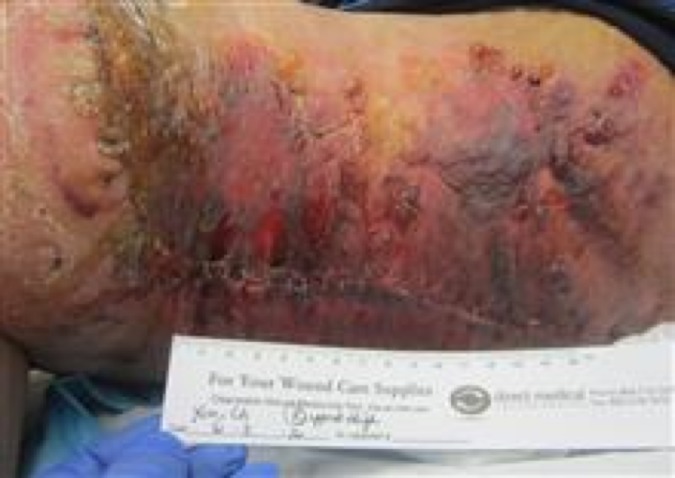
Failed Flap, Non-Healing Surgical Wound The figure shows the wound at the initial wound care clinic visit, after excision of right upper thigh lymphedema mass and failed surgical flap. Necrotic tissue and hematomas are noted. The area was painful for the patient upon arrival to the outpatient wound care center.

Two and one-half weeks later, the patient was referred to the Advanced Wound Care Center by his surgeon. The patient was seen and evaluated for a non-healing, necrotic, failed flap and surgical site. The patient, accompanied by his mother, arrived in a wheelchair to the visit. The wound area was severely necrotic, edematous with areas of scattered hematomas and blistering. The wound area was moderately warm to the touch with areas along the incision line of small dehiscence and draining serosanguinous and necrotic/brown exudate. The patient described the wound area as having intermittent sharp pains with occasional throbbing pain and burning sensations. The affected area measurements were 31 cm length x 21 cm width x 0.1 cm depth (Figure 1).

**Figure 2:**
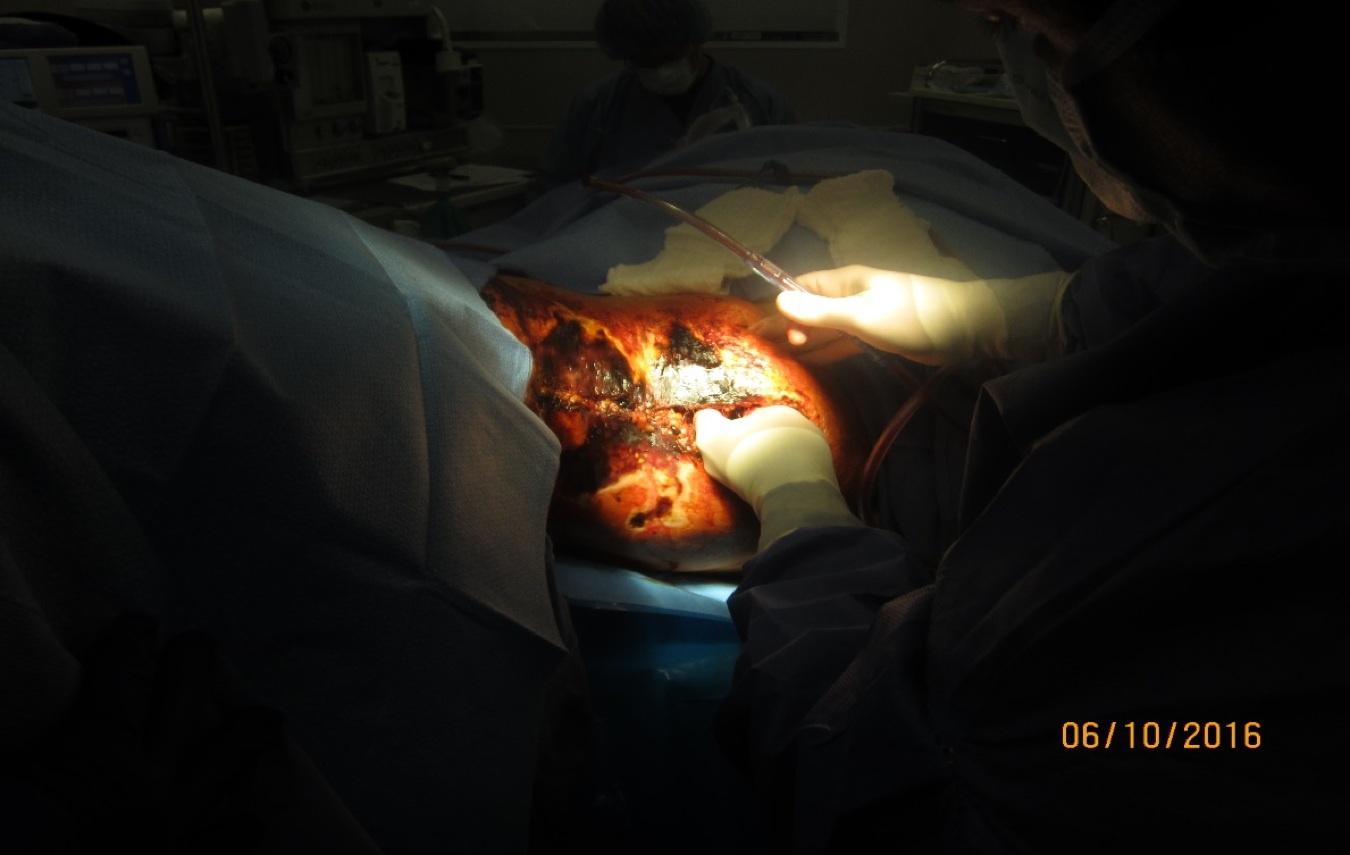
Surgical Debridement Continues on the Right Upper Thigh Wound A large undermining area was present, which also contributed to the wound's non-healing status. Necrotic and hematomas were noted deeper in the unhealthy tissue, which was removed and debrided by the surgeon.

Additional medical problems consisted of chronic obstructive pulmonary disease (COPD), asthma, hypertension, lymphedema, and venous insufficiency. The patient does not smoke, consume alcohol, or use illicit drugs. The patient is not a diabetic. Figure 2 illustrates the condition of the failed postoperative flap on the initial wound care clinic visit. Necrotic tissue is noted in the figure with poor postoperative non-healing.

**Figure 3:**
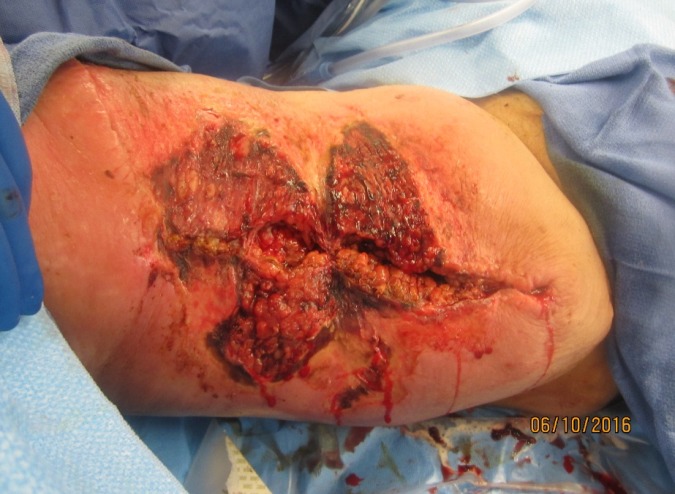
Completed Surgical Debridement of Right Upper Thigh Wound The surgical debridement was complete, presenting a cleaner wound bed. Part of the debridement was full-thickness, extending into the subcutaneous area. Superficial debridement was also performed to remove non-viable tissue. The hospital's Wound Care advanced practice registered nurse (APRN), was present in surgery and applied the VeraFlo™ instillation and dwell time negative pressure wound vac. Settings were: 18 minutes soak and dwell time of normal saline, every 2.5 hours with 125 mm/hg of constant pressure.

The flap area continued to quickly deteriorate, even from the time the surgeon had referred the patient to the wound care center until he was first seen. The patient was quickly sent for surgical debridement of the right thigh with a dehiscent surgical wound with non-MRSA staph cellulitis. After surgically removing eschar and necrotic tissue, the full thickness wound measured 18.0 cm x 21 cm width x 4.0 cm depth, with an undermining area of 14.0 cm at 12:00 – 2:00 and 6.5 cm at 4:00 – 6:00. During the surgery for wound debridement, necrotic tissue was excised from the non-healing wound on the right upper anterior/medial thigh region (Figure 3).

**Figure 4:**
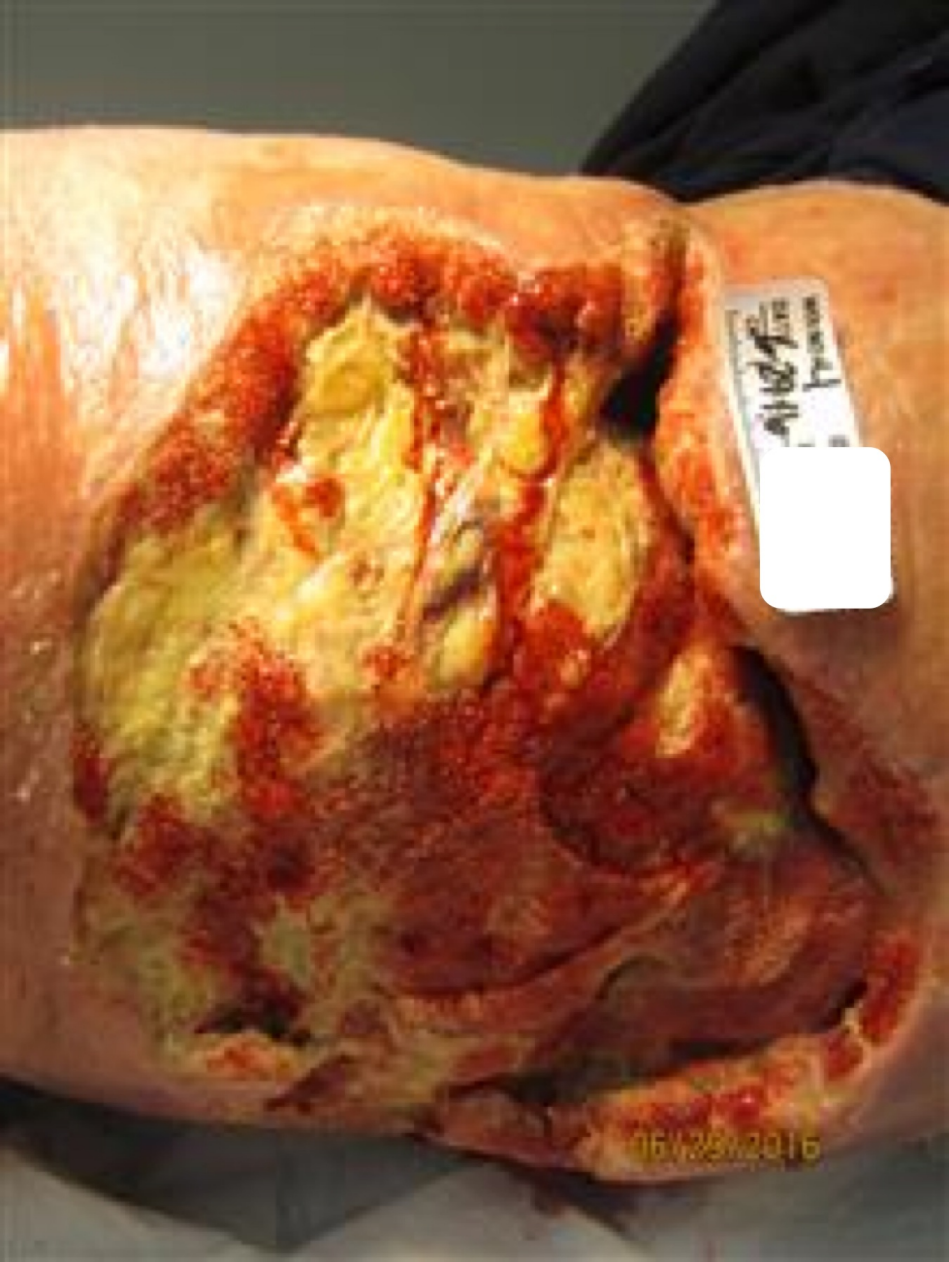
Right Upper Thigh Wound Three Weeks Postop The patient returned to the outpatient wound care clinic for his initial follow-up after surgical debridement, discharged from the hospital and the long-term acute care facility. The VeraFlo™ therapy consisted of almost three weeks of treatment, which continued cleaning and stimulating the wound. Red, beefy healthy granulation tissue formed, filling in the deep areas with healthy tissue. A regular wound vac was placed.

Figure 4 shows the removal of hematomas and the presence of undermining in surgery.

**Figure 5:**
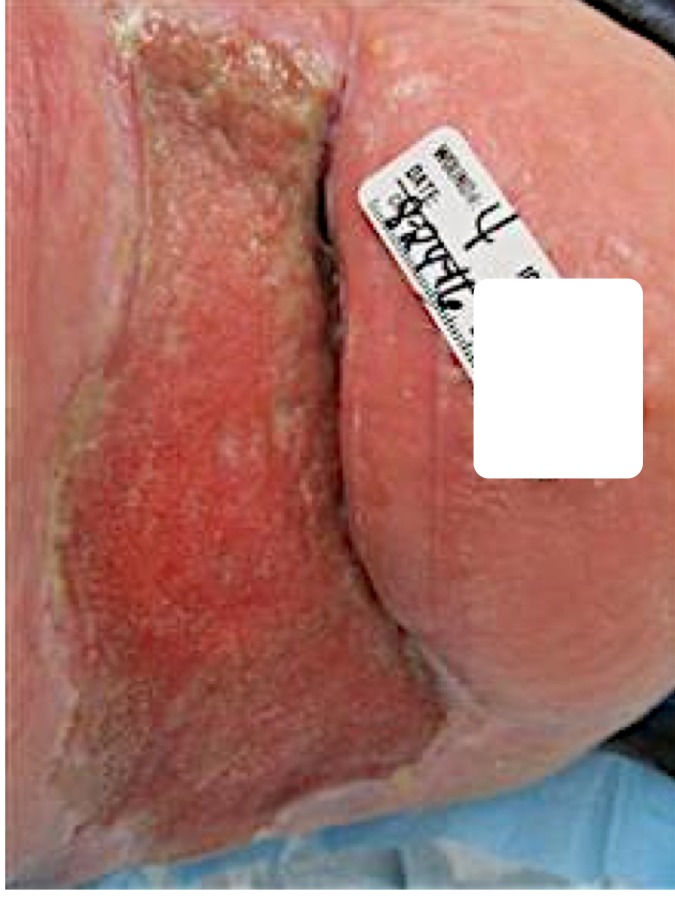
Right Upper Thigh Wound - One Month, Three Weeks Into Healing One month and three weeks after surgical debridement the wound is healing and healthy. Measurements are 10.5 cm length x 22.5 cm width x 1.8 cm depth. Granulation and epithelization tissue continues to form with no complications.

Figure 5 portrays the final state of the wound during surgical debridement. The wound vac dressing with normal saline was applied to the wound for treatment after debridement.

Laboratory results revealed WBC 8.1, RBC 3.63, HGB 9.4, HCT 30.2, neutrophils 3.53, lymphocytes 1.87, pre-albumin 10.9, sodium 136, potassium 3.4, ESR 83, and CRP 106. The right lower extremity venous insufficiency mixed with lymphedema 3+ and left lower extremity, also venous insufficiency mixed with lymphedema 1+. Skin changes had occurred on both the lower extremities with hard epidermis/lipodermatosclerosis and venous eczema. Testing for deep vein thrombosis was negative. Bilateral segmental pressures were unattainable due to the severity of the patient’s venous insufficiency and lymphedema disease. The arteries were severely calcified and non-compressible. The patient was admitted and the VeraFlo™ negative pressure treatment (Acelity L.P. Inc., San Antonio, TX) continued. The recommended dressing changes initially are 48 hours after beginning the treatment, then changes three times a week [3-4]. Infected wounds need to be monitored closely and changed more frequently than 48 to 72 hours than non-infected wounds [7-8].

Debridement is the removal of devitalized or non-living/necrotic tissue or exudate from a wound. Variations of debridements include surgical/sharp, biosurgical, mechanical, chemical, enzymatic, and autolytic [8].The wound was assessed by both the surgeon and the wound care APRN, who agreed on utilizing NPWTi-d. The solution of normal saline and the settings were decided by the wound care APRN. The mechanism of action is to remove edema, cellular debris, metabolic waste, and wound exudate, followed by wound cleansing, wound hydration, removing biofilm, and microstraining the tissue, which achieves a shorter healing time due to decreased irritants, such as excess matrix metalloproteases (MMPs) [10-11].

Concerns for repeated wound healing failure were partly due to the patient weighing 389 pounds with a BMI of 52.7, with chronic lymphedema applying pressure on the surgical site. While in the hospital, the patient was placed on a continuous air flow bariatric bed. He was assessed by the dietitian due to a low pre-albumin level and decreased wound healing. The patient was given daily a multiple vitamin, zinc, 220 mg, and vitamin C, 500 mg BID, along with protein supplementation. Nutrition is instrumental in successful wound healing. It is essential to meet the increased nutritional and metabolic needs of individuals with wounds. These appropriate caloric needs can be met with carbohydrates, protein, and fat requirements. Acceptable energy requirements of (30-35 calories/kg/day) is the essential recommended amount [13]. Foods that supply amino acids, such as meat, fish, poultry, dairy, and dried beans, should be the primary food source to promote collagen synthesis. Amino acids, such as L-arginine and glutamine, are recommended for large wounds. Hydration is also another essential component of healing because of fluid loss from draining wounds; without cardiac or renal compromise, the requirements are 30 ml/kg/day for adults. It is important to understand an obese person may be severely malnourished with a body mass index of greater than 35, especially with edema and infection. Micronutrients, such as vitamins and minerals (vitamins K, E, A, C, zinc, and copper), have a significant role in healing [14].It is essential to provide adequate education so that the patient fully understands the plan of care in all aspects, from the care of the wound to nutrition, diet, and supplements.

Lymphedema is a chronic, non-curable disorder. Primary lymphedema is the anomalous development of the lymphatic system or secondary lymphedema due to an injury of the lymphatic vasculature system [13]. Chronic lymphedema causes interstitial fluid to increase in the tissue, leading to fibrosis/hardening of the skin/tissues, chronic inflammation, fat disposition, and immense hypertrophy. Obesity increases the risk of secondary lymphedema. In fact, super obese people can develop lymphedema without surgery or injury, which is sometimes the cause of the damaged lymphatic system, resulting in lymphedema. The patient had suffered for approximately 15 years with chronic lymphedema in his bilateral lower extremities, which progressively worsened. The right upper medial thigh was the worst affected area of his lymphedema condition due to the severe size of the pannus, which extended down to the knee. Massive localized lymphedema is a term described being a benign soft tissue area which presents as a large mass in morbidly obese patients/adults. Chronic lymph flow is obstructed and/or disrupted, with drainage to the fat accumulation in the morbidly obese. Generally, the effective areas are located in the lower extremities. Common areas affected are the upper medial thighs but can also be in the anterior and posterior thigh regions. Generally, the mass is painless but may affect the patient’s physical, social, and psychological well-being [15].

The patient was seen the next day with adequate pressure/suction maintained with minimal serosanguineous drainage in the canister. Normal saline was continued as the instillation fluid with no changes to the settings. The patient was daily placed on supplements for healing, a multiple vitamin with minerals, zinc, 220 mg, and vitamin C, 500 mg twice a day. The dietitian also visited the patient for an adequate protein supplement. Physical therapy was consulted for strengthening and mobilization. Tubigrip™ (Mölnlycke Health Care, Göteborg, Sweden) size F compression stockings were applied to the bilateral lower extremities.

**Figure 6:**
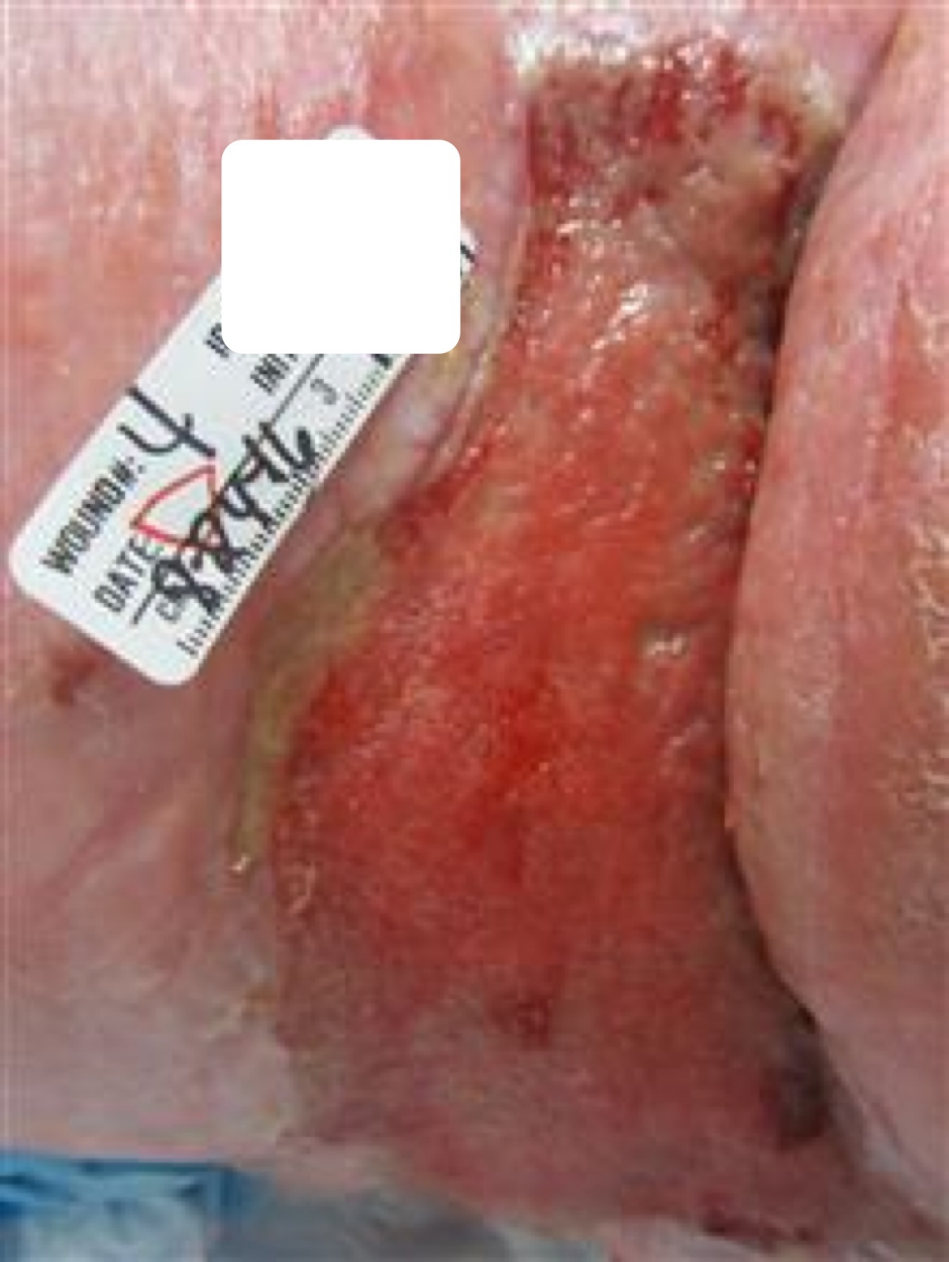
Right Upper Thigh Wound - Two Months and One Week Into Healing Last picture of the wound to date, two months and one week after original surgical debridement. Measurements are 7.5 cm length x 20.5 cm width x 0.1 cm depth. Continued granulation and epithelization tissue with the continuation of negative pressure therapy.

Two days after the surgery and NPWTi-d placement, the patient was seen by the Wound Care APRN. The patient was pre-medicated for pain control for the subsequent dressing change. Unfortunately, some dressing changes can be uncomfortable and challenging. The patient was pre-medicated with an opioid analgesic for pain relief. A non-sting adhesive removal pad was used to loosen the dressing, which decreased discomfort from the adhesiveness of the drape. Lidocaine 4% liquid topical solution, 50 ml, was instilled onto the foam for additional pain control when the black foam was removed [16]. Soak time of the lidocaine was approximately 20 minutes before removal of the black foam, which definitely reduced pain when the foam was removed from the wound bed. At discharge, the patient’s wound reflected improvements with a cleaner, healthier wound bed. Red granulation tissue was forming. The patient was discharged to an LTAC (long-term acute care facility) with recommendations to continue his current treatment. The NPWTi-d was continued for the next several weeks. The patient was seen after the LTAC discharge two and one-half weeks later in the wound care center for continued care and treatment with improved wound healing as seen in Figure 6. Smaller measurements were noted and granulation and epithelization tissue were developing.

## Discussion

After the LTAC discharge and subsequent follow-up visit at the Advanced Wound Care Clinic (three weeks after surgical debridement), his wound was improving with no clinical signs and symptoms of deterioration. Measurements were 16.5 cm length x 23.5 cm width x 3.1 cm depth. The patient felt better and was able to better ambulate. A regular NPWT (negative pressure wound therapy) was placed after the discharge from the LTAC and when received back to the Advanced Wound Healing Center. The patient received weekly debridements with the removal of non-vital tissue, which provided a cleaner, more prepared wound bed for tissue growth. Nutrition and supplements continued with improvements in his treatment. The patient’s pre-albumin level upon returning to the Advanced Wound Healing Center had increased to an improved level of 18.7, with decreasing levels of ESR - 8 and CRP - 26. The wound was negative for bacterial growth. The wound continued with good healing.

The NPWT continued as the treatment of choice with dressing changes three times a week, increased protein intake, and vitamin supplements; the wound had continued to improve with the development of granulation and epithelization tissue, with pink and healthy tissue. This is noted in Figure 6, which is the last picture taken to date in the wound care outpatient center.

It is recommended by the author to implement these combined treatments as soon and as early as possible when encountering difficult wounds. Despite difficulties with continued off-loading due to obesity and lymphedema affecting the wound area, the patient has continued to improve. There have been no further hospitalizations, surgery, or IV antibiotics, and the wound healing trajectory time is expected soon, thus all decreasing costs. It is worthy to say that these savings all stem from the early and continued NPWTi-d usage, placed directly at the time of the surgical debridement and the continuation into the LTAC setting. The author recommends the usage of the NPWTi-d with saline for the treatment of difficult wounds. This therapy allowed the ongoing removal of large amounts of necrotic tissue, bacteria, bio-burden, debris, and exudate, enabling the wound onto the path of continued healing [16]. The user/provider had the choice of a variety of instillation solutions as listed in Table 3.

**Table 3:** Compatible Solutions With VeraFlo™ Instillation VAC Therapy The following solutions are recommended by KCI, the VeraFlo™ manufacturer [12].

Normal Saline
Hypochlorite-based solution (hypochlorous acid, sodium hypochlorite, or ¼ strength Dakin’s solution (label container "for wound vac use only")
Lactated Ringer’s solution
Silver nitrate (0.5%)
Biguanides (polyhexanide, such as Prontosan® and Lavasept®)
Sulfur-based solutions (mafenide acetate/ Sulfamylon®)
Cationic solutions (octenidine and benzalkonium chloride, such as Octenilin® and Zephiran®) [2].

## Conclusions

Chronic wounds continue to be a barrier in wound healing. Several factors can cause a wound to deteriorate and stall. Wound cleansing is a decisive step in proper wound management. Utilizing the best regime is fundamental in preparing the wound bed by removing elevated inflammatory and enzymatic elements, cellular debris, necrotic tissue, and infection. Implementing the NPWTi-d provides the cleansing of devitalized tissue, exudates, debris, and infection, leaving cleaner healthier wound tissue.

Granulation and epithelization tissue were present, with accounted improvements due to the instillation of normal saline via the instillation and dwell time option. The NPWTi-d uses black foam with reticulated open cell foam technology dressings. This allows the foam dressing to have a more even distribution of fluid in the wound [11]. Debridements are essential to keep the wound bed clean and prepared for treatment and healing. The healing effects of the saline instillation were enhanced due to the clean wound bed preparation [16].

Antibiotics are an essential treatment for infected or complex wounds, such as surgical wounds, colonized wounds, postoperative dehiscence, or soft tissue wounds [10]. NPWTi-d with normal saline was effective in the treatment of Staphylococcus aureus, which was present in our patient’s wound.

The lymphedema pannus was surgically debrided to improve the patient's overall health and status. The NPWTi-d option was chosen for the primary treatment due to an increased need of granulation tissue, quicker wound closure, and decreasing the chance of reinfection. Also, antibiotics, nutrition, supplements, and therapies played an important role in the patient's improvement. Significant quicker healing occurred for the patient, resulting in easier attention to personal needs, improved work, social status, and quality of life. It is the conclusion of the author that the increased healing rate, with no re-hospitalizations or further surgical debridements, was due to the early treatment and ongoing NPWTi-d. Costs were decreased due to ongoing wound healing with no further complications. Thanks to all the continued past research and developments, this advanced treatment has been shown and proven to be a valuable treatment in the advancement of wound healing [16].
